# Obstructive Sleep Apnea and Type 2 Diabetes: Is There a Link?

**DOI:** 10.3389/fneur.2012.00126

**Published:** 2012-08-13

**Authors:** Sushmita Pamidi, Esra Tasali

**Affiliations:** ^1^Respiratory Division, Department of Medicine, McGill UniversityMontreal, QC, Canada; ^2^Department of Medicine, The University of ChicagoChicago, IL, USA

**Keywords:** sleep apnea, diabetes, glucose, insulin, CPAP, cardiovascular

## Abstract

Type 2 diabetes is a chronic illness that is increasing in epidemic proportions worldwide. Major factors contributing to the development of type 2 diabetes include obesity and poor lifestyle habits (e.g., excess dietary intake and limited physical activity). Despite the proven efficacy of lifestyle interventions and the use of multiple pharmacological agents, the economic and public health burden of type 2 diabetes remains substantial. Obstructive sleep apnea (OSA) is a treatable sleep disorder that is pervasive among overweight and obese adults, who represent about two thirds of the U.S. population today. An ever-growing number of studies have shown that OSA is associated with insulin resistance, glucose intolerance and type 2 diabetes, independent of obesity. Evidence from animal and human models that mimic OSA provides potential mechanisms for how OSA may alter glucose metabolism. Up to 83% of patients with type 2 diabetes suffer from unrecognized OSA and increasing severity of OSA is associated with worsening glucose control. However, it is still unclear whether OSA may lead to the development of diabetes over time. More data from large-scale longitudinal studies with rigorous assessments of diabetes and OSA are needed. In addition, there is still controversy whether continuous positive airway pressure (CPAP) treatment of OSA improves glucose metabolism. Large-scale randomized-controlled trials of CPAP treatment of OSA with well-validated assessments of insulin sensitivity and glucose tolerance are needed. These studies may reveal that OSA represents a novel, modifiable risk factor for the development of prediabetes and type 2 diabetes.

Obstructive sleep apnea (OSA) is a treatable sleep disorder that is pervasive among overweight and obese adults, who represent about two-thirds of the U.S. population today. Prevalence estimates of OSA in obese adults (aged 30–69 years) range from 11 to 46% in women and 33 to 77% in men (Young et al., 2005). Moreover, weight gain longitudinally predicts increased OSA incidence and severity (Peppard et al., 2000; Newman et al., 2005).

Over the past decade, there has been a rising interest in the cardiometabolic derangements associated with OSA (reviewed in Punjabi and Polotsky, 2005; Somers et al., 2008; Tasali and Ip, 2008; Tasali et al., 2008b; Jun and Polotsky, 2009; Levy et al., 2009). Current data suggests that OSA may play a role in adverse cardiovascular outcomes (Yaggi et al., 2005; Young et al., 2008; Redline et al., 2010). A large number of studies have shown that OSA is associated with insulin resistance, glucose intolerance, and type 2 diabetes, independent of obesity. In this article, we will review the current evidence from studies that address frequently asked questions regarding the links between OSA, type 2 diabetes, and prediabetic states.

## What is the Natural History of Type 2 Diabetes?

Type 2 diabetes is a chronic illness that is increasing in epidemic proportions worldwide. It affects 25.6 million adults in the U.S., with an estimated 1.9 million new diagnoses per year [Center for Disease Control (CDC), 2011]. Major factors contributing to the development of type 2 diabetes include obesity and poor lifestyle habits (e.g., excess dietary intake and limited physical activity). The current standard criteria for the diagnosis of type 2 diabetes are as follows: (1) Hemoglobin A1c (A1c) ≥6.5%, or (2) fasting plasma glucose ≥126 mg/dl, or (3) 2 h plasma glucose ≥200 mg/dl during an oral glucose tolerance test (OGTT), or (4) random plasma glucose ≥200 mg/dl in a patient with classic symptoms of hyperglycemia [American Diabetes Association (ADA), 2010].

High glucose levels not sufficient to meet the diagnostic criteria for type 2 diabetes are considered as prediabetic states, namely impaired fasting glucose (IFG) and impaired glucose tolerance [IGT; American Diabetes Association (ADA), 2010]. According to the CDC, 35% of U.S. adults aged 20 years or older may have prediabetes, which suggests that prediabetic states exist in an estimated 79 million Americans [Center for Disease Control (CDC), 2011]. Fasting plasma glucose levels that are in the range of 100–125 mg/dl define an IFG state. A 2-h plasma glucose level during the 75-g OGTT that is in the range of 140–199 mg/dl defines an IGT state. In prediabetic states, the A1c ranges between 5.7 and 6.4% [American Diabetes Association (ADA), 2010]. The hallmark of prediabetic states is insulin resistance. Transition from the early states of impaired glucose metabolism to frank diabetes may take several years, and it is estimated that ∼70% prediabetic individuals eventually develop type 2 diabetes (Nathan et al., 2007). The most commonly used clinical and research methods to assess glucose tolerance, insulin sensitivity, and secretion are summarized in Table 1.

**Table 1:** Various methods used in the assessment of glucose metabolism*.

Method	Description	Reliability and interpretation
Fasting plasma glucose (FPG) and insulin	Plasma glucose and serum insulin levels are measured in a fasting blood sample	Impaired fasting glucose (IFG) is diagnosed if FPG is between 100 and 125 mg/dl
		Diabetes is diagnosed if fasting glucose levels are ≥126 mg/dl [American Diabetes Association (ADA), 2010]
Hemoglobin A1c (A1c)	Measured in a single blood sample and reflects glucose control over the preceding 2–3 months	Diabetes if hemoglobin A1c ≥6.5% Prediabetes if hemoglobin A1c is between 5.7 and 6.4% [American Diabetes Association (ADA), 2010]
		Monitoring of hemoglobin A1c is used in clinical practice for diabetes management and is the primary target for glycemic control
		Lowering hemoglobin A1c below 7% has been associated with a reduction of diabetic complications [American Diabetes Association (ADA), 2010]
Homeostatic model assessment (HOMA) index	The normalized product of fasting glucose and insulin calculated using the following formula: (fasting serum insulin × fasting plasma glucose)/22.5	Reliable and validated estimate of insulin resistance (Matthews et al., 1985)
		Elevated HOMA levels reflect higher degrees of insulin resistance
Oral glucose tolerance test (OGTT)	After ingestion of 75 g of glucose, blood samples are collected for the measurement of glucose and insulin concentrations at time 30, 60, 90, and 120 min to evaluate glucose tolerance	A clinical tool used for the diagnosis of type 2 diabetes [American Diabetes Association (ADA), 2010]
		Normal glucose tolerance (NGT), impaired glucose tolerance (IGT), or diabetes is diagnosed if the glucose level at 2 h is less than 140 mg/dl, between 140–199 mg/dl, or 200 mg/dl or more, respectively
Continuous glucose monitoring system	Glucose concentration in the interstitial fluid is measured using a subcutaneous sensor attached to a continuous monitoring device that record sensor signals every 5 min, providing 288 glucose level readings per day	Used in clinical practice for diabetes management to assess 24 h glucose fluctuations (particularly post-prandial and nocturnal levels)
Hyperinsulinemic euglycemic clamp	Insulin sensitivity is quantified by intravenous glucose infusion rate (i.e., glucose uptake by all the tissues in the body) under steady state conditions of euglycemia	The gold standard technique used for measurement of insulin sensitivity (DeFronzo et al., 1979)
Intravenous glucose tolerance test	Glucose and insulin concentrations are measured during fasting and after intravenous glucose injection at frequent intervals for 4 h	Validated tool that allows to simultaneously assess glucose tolerance, beta-cell responsiveness, and insulin sensitivity using a mathematical model (Bergman, 2005)

*Reprinted with permission of the American Thoracic Society. Copyright © 2008 American Thoracic Society.

*Tasali and Ip (2008), An official Journal of The American Thoracic Society*.

In normal individuals, insulin resistance is compensated by an up-regulation of insulin secretion to maintain normal glucose tolerance. According to the “hyperbolic law of glucose tolerance,” under normal conditions the product of insulin sensitivity and secretion (i.e., the disposition index; DI) is constant (Bergman, 2005). In individuals who are at risk for developing type 2 diabetes, the DI may be reduced due to inadequate compensatory insulin secretion for a given degree of insulin resistance. Thus, type 2 diabetes is a two-step process characterized by both insulin resistance and impaired insulin secretion. Initially, insulin resistance occurs resulting in progression from a normal glucose tolerant state to IGT. At this stage, insulin levels are elevated (compensatory hyperinsulinemia) and beta-cell dysfunction is already present. Subsequently, progressive decline in beta-cell function occurs, which leads to development of type 2 diabetes (DeFronzo and Abdul-Ghani, 2011).

Despite the proven efficacy of lifestyle interventions and the use of multiple pharmacological agents, the economic and public health burden of type 2 diabetes remains substantial. Cardiovascular disease is a major cause of mortality and morbidity in both type 2 diabetes and prediabetes. In addition to cardiovascular disease, type 2 diabetes is associated with longterm complications affecting the eyes, kidneys, and nervous system. (Kashyap and Defronzo, 2007; Nathan et al., 2007; Mazzone et al., 2008). It is well documented that both lifestyle modification and metformin use are effective in reducing the incidence of diabetes (Tuomilehto et al., 2001; Knowler et al., 2002, 2009). However, even with substantial efforts to achieve lifestyle modification, data suggest that the effectiveness of this intervention diminishes over time. Thus, the development of additional strategies to prevent or delay the onset of diabetes and its cardiovascular complications remains a critical public heath challenge.

## What is the Prevalence of OSA in Type 2 Diabetics?

The overall prevalence of OSA diagnosed by full polysomnography in type 2 diabetic patients is approximately 71% based on the average of data from five studies including a total number of nearly 1200 type 2 diabetic patients (Resnick et al., 2003; Einhorn et al., 2007; Foster et al., 2009; Laaban et al., 2009; Aronsohn et al., 2010; Table 2). The highest estimate of 86% was reported in obese diabetic patients enrolled in the multi-center Sleep AHEAD study (Foster et al., 2009). The lowest estimate of 58% was found in the Sleep Heart Health Study (Resnick et al., 2003), which is a multi-center cohort that included older individuals (with more than half aged over 65 years) and used self-reported diabetes and a desaturation criteria of 4% for the definition of hypopneas. In another study by Aronsohn et al., OSA prevalence was found to be 77% using a less stringent cut-off of 3% for oxygen desaturations. Of note, when the dataset was re-analyzed using more strict 4% desaturation criteria, this estimate was reduced to 58% (Aronsohn et al., 2010). These variable prevalence estimates may be due to differences in study populations as well as different scoring criteria used to capture OSA (Redline et al., 2000). Nevertheless, considering that the prevalence of OSA by full-night polysomnography in patients with type 2 diabetes averages 71%, this would then suggest that approximately 19 million diabetics may have undiagnosed and untreated OSA. Indeed, a recent retrospective analysis of a total of 16,066 diabetic patients with one or more primary care office visits in 27 primary care ambulatory practices has revealed that only 18% of the study population (23% of obese patients) carried an OSA diagnosis (Heffner et al., 2012). These findings suggest that in the primary care setting, OSA is largely unrecognized in patients with diabetes and thus millions of diabetics may currently suffer from this co-morbidity. In sum, these staggering statistics emphasize the need for a better understanding of the links between OSA and type 2 diabetes (Shaw et al., 2008).

**Table 2:** **Studies examining the prevalence of obstructive sleep apnea (OSA) in patients with type 2 diabetes**.

Author/year	Sample characteristics	OSA diagnosis criteria	Diabetes definition	Prevalence estimates
Resnick et al. (2003)	*n* = 470 (216 men) Sleep Heart Health Study, USA	AHI ≥5	Self-reported; Use of diabetes medications	58% with at least mild OSA (AHI ≥5) 24% with moderate-severe OSA (AHI ≥15)
Einhorn et al. (2007)	*n* = 62 (31 men) Diabetes clinics	AHI ≥5	Not reported	71% with at least mild OSA (AHI ≥5) 61% with moderate-severe OSA (AHI ≥15)
Foster et al. (2009)	*n* = 305 (122 men) Obese patients with type 2 diabetes enrolled in Sleep AHEAD trial	AHI ≥5	Self-reported with verification	86.6% Overall prevalence 33.4% Mild OSA 30.5% Moderate OSA 22.6% Severe OSA
Laaban et al. (2009)	*n* = 303 (155 men) Poorly controlled type 2 diabetics from clinics	AHI ≥5	Documented medical history of diabetes	63% Overall prevalence 34% Mild OSA 19% Moderate OSA 10% Severe OSA
Aronsohn et al. (2010)	*n* = 60 (27 men) Primary care and endocrinology clinics	AHI ≥5	Physician diagnosis	77% Overall prevalence 38% Mild OSA 25% Moderate OSA

## What is the Prevalence and Incidence of Type 2 Diabetes in OSA?

Several population and clinic-based studies have assessed the prevalence and incidence of type 2 diabetes in OSA (Meslier et al., 2003; Reichmuth et al., 2005; Seicean et al., 2008; Tamura et al., 2008; Botros et al., 2009; Mahmood et al., 2009; Marshall et al., 2009; Ronksley et al., 2009; Celen et al., 2010; Fredheim et al., 2011). The majority of these studies have used validated diabetes definitions based on fasting glucose or 2 h post-challenge glucose levels, while a few studies relied on self-reported diabetes and/or antidiabetic medication use. Most, but not all studies (Marshall et al., 2009; Ronksley et al., 2009; Celen et al., 2010), used full polysomnography to diagnose OSA. The majority of studies used statistical adjustments for shared risk factors such as age, sex, body mass index (BMI) and various other parameters (e.g., neck and waist circumference). Overall, the cross-sectional analyses have shown a significantly higher prevalence of diabetes in patients with OSA as compared to those without OSA. The prevalence estimates range from 15 to 30%, depending on the study population, the definition of OSA severity, and the methods used to diagnose type 2 diabetes (Meslier et al., 2003; Reichmuth et al., 2005; Mahmood et al., 2009; Ronksley et al., 2009; Fredheim et al., 2011). A few studies have also reported a significant relationship between increasing severity of OSA and the prevalence of diabetes (Reichmuth et al., 2005; Tamura et al., 2008; Ronksley et al., 2009; Samson et al., 2012). In two studies (Mahmood et al., 2009; Marshall et al., 2009), the higher odds ratio for diabetes in patients with OSA was no longer significant after controlling for confounders. In one study (Ronksley et al., 2009), a higher adjusted odds of self-reported diabetes was found only in the severe OSA group who had excessive sleepiness. In this regard, more research is needed to elucidate the role of sleepiness in adverse metabolic outcomes and to better understand the links between OSA and diabetes in sleepy versus non-sleepy patients.

A total of five prospective studies have examined the incidence of type 2 diabetes in patients with OSA (Table 3). In the longitudinal follow-up of about 1000 individuals from the Wisconsin Sleep Cohort (Reichmuth et al., 2005), OSA was found to be a risk factor for incident diabetes over a 4-year period, but this association was no longer significant after adjusting for age, sex, and body habitus. The Busselton Health Study (Marshall et al., 2009) reported a significant independent association between moderate to severe OSA and incident diabetes over a 4-year follow-up period, but the sample size was small with only a few incident cases of diabetes. In another study by Botros et al. (2009), an independent association between OSA and incident diabetes was found after adjusting for age, sex, race, BMI, baseline fasting glucose and weight change over a mean follow-up period of 2.7 years. Interestingly, one clinic-based study of 168 middle-aged patients (Celen et al., 2010), reported a significantly higher incidence of diabetes in patients with OSA compared to those without OSA in women, but not in men, after a follow-up period of 16 years. More recently, in a population-based cohort of 156 men from Sweden (Lindberg et al., 2012), the increasing severity of oxygen desaturations in OSA was found to be a predictor of developing diabetes after adjustments for various confounders including years of treatment for OSA. In another prospective study of about 4000 middle-aged individuals, nocturnal intermittent hypoxia was associated with an increased risk of developing type 2 diabetes after a 3-year median follow-up (Muraki et al., 2010).

**Table 3:** **Prospective studies examining the incidence of type 2 diabetes in patients with obstructive sleep apnea (OSA)**.

Author/year	Sample characteristics	OSA diagnosis criteria	Diabetes definition	Follow-up period	Main findings
Reichmuth et al. (2005)	1387 (779 men); Wisconsin Sleep Cohort, USA	AHI ≥5	Physician diagnosis; Fasting glucose ≥126 mg/dl	4 years (*n* = 987)	In unadjusted analysis, higher incidence of diabetes in moderate to severe OSA. Unadjusted OR = 4.06 (CI = 1.86–8.85)
					After adjustments for age, sex, body habitus, no increase in diabetes incidence. Adjusted OR = 1.62 (CI = 0.67–3.65)
Marshall et al. (2009)	399 (294 men); Busselton Health Study, Australia	AHI ≥5 (limited PSG)	Physician diagnosis; Use of diabetes medications; Fasting glucose ≥126 mg/dl	4 years	After adjustments for age, sex, BMI, waist, mean blood pressure, HDL cholesterol, higher incidence of diabetes in moderate to severe OSA. Adjusted OR = 13.45 (CI = 1.59–114.11)
Botros et al. (2009)	544 individuals; VA Connecticut Sleep Center, USA	AHI ≥8 (full PSG)	Physician diagnosis; Fasting glucose ≥126 mg/dl	2.7 years	After adjustments for age, sex, race, BMI, baseline fasting glucose, weight change, higher incidence of diabetes with increasing severity of OSA. Adjusted Hazard Ratio per quartile of OSA severity = 1.43 (CI = 1.10–1.86)
Celen et al. (2010)	168 middle-age adults, sleep clinics, Sweden	OD ≥30 (limited PSG)	Physician diagnosis	16 years	Significantly higher incidence of diabetes in patients with OSA compared to no OSA only in women but not in men
Lindberg et al. (2012)	156 men, population-based cohort, Sweden	AHI >5	Self-reported; Regular doctor visits for diabetes; Fasting glucose ≥126 mg/dl	11 years	At the follow-up, 23 men had diabetes After adjustment for age, baseline BMI, change in BMI, hypertension, and years with CPAP, increasing severity of oxygen desaturations is a predictor of developing diabetes. Adjusted OR = 4.4 (CI = 1.1–18.1)

*AHI, apnea-hypopnea index; CPAP, continuous positive airway pressure; OD, oxygen desaturations*.

Thus, the current evidence from cross-sectional analyses suggest that type 2 diabetes is more prevalent among patients with OSA compared to those without OSA, independent of shared risk factors. However, it is still unclear whether OSA may lead to the development of diabetes over time. More data from large-scale longitudinal studies with rigorous assessments of diabetes and OSA are needed.

## Does Untreated OSA Adversely Affect Glycemic Control in Type 2 Diabetic Patients?

An earlier study including 279 diabetic patients found no significant associations between OSA and glycemic control, as assessed by A1c (Einhorn et al., 2007). In this negative study, only about 22% of the study population was assessed by full polysomnography and the duration of sleep recording was reported to be as low as 4 h. In a more recent study (Aronsohn et al., 2010), 60 patients with physician-diagnosed diabetes underwent full laboratory polysomnography (with a minimum laboratory recording time of 7 h) to assess the presence and severity of OSA. Increasing severity of untreated OSA was associated with poorer glucose control after controlling for age, sex, race, BMI, number of diabetes medications, level of exercise, years of diabetes, and total sleep time. Compared to patients without OSA (adjusted mean A1c: 5.7%), the adjusted mean A1c was 7.3% in mild OSA, 7.7% in moderate OSA, and 9.7% in severe OSA. Importantly, the magnitude of these associations is comparable, if not exceeding to those of widely used hypoglycemic medications. Of note, when only the first 4 h of recording was analyzed, the robust relationship between OSA severity and A1c was no longer apparent, perhaps due to insufficient recording time for REM sleep to occur. This would suggest that sleep recordings longer than the commonly used minimum of 4 h might be needed for better assessment of the links between OSA and type 2 diabetes. Another cross-sectional study (Pillai et al., 2011) involving 52 consecutive patients recruited from a diabetes-obesity clinic found that after adjusting for age, gender, BMI, duration of diabetes, and insulin dose, increased severity of OSA was associated with increased A1c levels. The adjusted mean values of A1c in each OSA category were 8.62% for no OSA, 9.36% for mild, 10.61% for moderate, and 9.91% for severe OSA. In a smaller study (Fendri et al., 2011) of 26 overweight and obese individuals with type 2 diabetes, nocturnal glycemia (as assessed by a continuous glucose monitoring sensor) was found to be 38% higher in those who had OSA, compared to those without OSA, independent of BMI.

Overall, there is evidence to suggest that the presence and severity of untreated OSA may be associated with poor glucose control in type 2 diabetic patients. These findings may have important clinical implications in the management and care of type 2 diabetes as they support the hypothesis that reducing the severity of OSA may be an adjunct therapeutic strategy to optimize glucose control.

## Is the Presence and Severity of OSA Associated with Prediabetic States such as Insulin Resistance and Glucose Intolerance?

Over the past few decades, an increasing number of studies from population and clinic-based cohorts have consistently found a robust independent association between the presence and severity of OSA and prediabetic states such as insulin resistance and glucose intolerance. A comprehensive review of all these studies is beyond the scope of this article, but has been the topic of several review articles (Punjabi and Polotsky, 2005; Tasali and Ip, 2008; Jun and Polotsky, 2009; Levy et al., 2009; Punjabi, 2009; Pamidi et al., 2010). In the majority of these studies the estimation of insulin sensitivity was based on the homeostatic model assessment (HOMA) index, which is a normalized product of fasting glucose and insulin levels. Several studies have also used the OGTT to assess glucose and insulin response after glucose ingestion. Interestingly, many of these studies were conducted in men, so the data on possible gender effects in the associations between OSA, insulin resistance, and glucose intolerance is lacking. In the majority of studies, commonly used indices of OSA severity were the apnea-hypopnea index (AHI) and the frequency as well as the degree of intermittent hypoxia.

Overall, the prevalence of prediabetes (i.e., the presence of either IFG and/or IGT) was found to be significantly higher in patients with OSA than those without OSA, but the estimates have been variable, ranging between 20 and 67% (Meslier et al., 2003; Kono et al., 2007; Seicean et al., 2008; Tamura et al., 2008; Fredheim et al., 2011). In a large cross-sectional analysis of a subset of data from the Sleep Heart Health Study, involving over 2500 non-diabetic individuals (Seicean et al., 2008), the presence of OSA was associated with a significantly higher odds of IFG and IGT after controlling for age, sex, race, BMI, and waist circumference. Importantly, the magnitude of these associations was similar in non-overweight and overweight individuals (Seicean et al., 2008).

In the earliest studies from large population-based samples, OSA was found to be independently associated with insulin resistance (Ip et al., 2002; Punjabi et al., 2002) and glucose intolerance (Punjabi et al., 2002). In a clinic-based sample of 595 men, the increasing severity of OSA was associated with worsening glucose tolerance and insulin resistance, independent of age and BMI (Meslier et al., 2003). In an earlier report from the Sleep Heart Health Study, the increasing severity of OSA was independently associated with both fasting and 2 h post-load glucose levels during an OGTT (Punjabi et al., 2004). In a population-based sample of 400 women, those with severe OSA had significantly lower insulin sensitivity as compared to those without OSA, and the severity of OSA was associated with increasing fasting and 2-h post-challenge insulin levels during an OGTT, after adjusting for age, waist-hip ratio, and other potential confounders (Theorell-Haglow et al., 2006). In an analysis of a subset of participants in the Wisconsin Sleep Cohort Study (*n* = 546), OSA was significantly associated with insulin resistance as measured by the HOMA index (Nieto et al., 2009). More recently, Punjabi and Beamer (2009) estimated insulin sensitivity in 118 non-diabetic adults using a frequently sampled intravenous glucose tolerance test (ivGTT). The authors found that compared to normal individuals, those with mild, moderate, and severe OSA showed a 26.7, 36.5, and 43.7% decrease in insulin sensitivity, respectively, after controlling for age, gender, race, and percent body fat. Despite this progressive decline in insulin sensitivity, the acute insulin response to glucose was unchanged, suggesting an insufficient up-regulation of insulin secretion and thus impaired beta-cell function. A recent cross-sectional study of 1,599 non-diabetic patients, where participants with an A1c of ≥6.5%, self-reported diabetes or use of diabetic medication were excluded, a dose–response relationship between OSA severity and the percentage of patients with A1c >6% was observed, suggesting a gradual increase in prediabetic state with increasing severity of OSA (Priou et al., 2012). Although some of these studies have controlled for adiposity using several surrogate markers (e.g., waist circumference, percent body fat), central adiposity, particularly visceral fat is an important confounding factor in the observed links between OSA and prediabetic states. In this regard, there is some evidence to suggest that in the absence of confounding effects of visceral adiposity, OSA may still be associated with insulin resistance and higher fasting glucose levels (Kono et al., 2007).

A recent study by Pamidi et al. (2012), the researchers assessed whether the presence of OSA affects glucose metabolism in young, lean individuals who are healthy and free of cardiometabolic disease. In a prospective design, 52 healthy men (age range 18–30 years and BMI range 18–25 kg/m^2^) underwent a laboratory polysomnogram followed by a morning OGTT to assess glucose metabolism. All subjects were stratified according to the presence or absence of ethnicity-based diabetes risk and family history of diabetes. The authors then used a frequency matching approach and randomly selected individuals without OSA, yielding a total of 20 control men without OSA and 12 men with OSA. Men with OSA and controls were similar in terms of age, BMI, ethnicity-based diabetes risk, family history of diabetes, and level of exercise. Both groups had normal systolic and diastolic blood pressure and fasting lipid levels. Following ingestion of a 75-g glucose load, men with OSA had 27% lower insulin sensitivity (estimated by the Matsuda index during the OGTT) and 37% higher total insulin secretion than the controls, despite comparable glucose levels. This study is the first to demonstrate that in young lean men (mean age <24 years, mean BMI <23 kg/m^2^) who are otherwise healthy and free of cardiometabolic disease, the presence of OSA is associated with insulin resistance and compensatory hyperinsulinemia to maintain normal glucose homeostasis. These findings provide evidence to support the hypothesis that OSA may be associated with early changes in the natural history of type 2 diabetes even in the absence of obesity, cardiovascular disease, and other known risk factors for type 2 diabetes.

In summary, these observational studies strongly support an association between OSA and insulin resistance and glucose intolerance, even after statistical adjustments for shared risk factors. However, central adiposity, particularly visceral fat, needs to be considered as an important confounder when interpreting these studies. Finally, studies with longitudinal and interventional designs are needed to better address the potential role of OSA in the development of prediabetic states.

## Is CPAP Treatment of OSA Effective in Improving Glucose Metabolism?

### Evidence from uncontrolled studies

In patients with type 2 diabetes, five uncontrolled studies including a total of 87 patients examined the effects of continuous positive airway pressure (CPAP) on glycemic control (Brooks et al., 1994; Harsch et al., 2004; Babu et al., 2005; Dawson et al., 2008; Pallayova et al., 2008). Two studies reported improvements in nighttime glucose levels after one night (Pallayova et al., 2008) and 5 weeks (Dawson et al., 2008) of CPAP use. Two other studies showed improvements in insulin sensitivity (as assessed by the gold standard hyperinsulinemic euglycemic clamp), without a change in A1c levels after 3–4 months of CPAP (Brooks et al., 1994; Harsch et al., 2004). In contrast, Babu et al. (2005) found an improvement in A1c and post-prandial glucose levels after 3 months of CPAP therapy in 25 obese patients with type 2 diabetes. Notably, in patients who used CPAP for more than 4 h/night (averaging ∼6.6 h/night) the decrease in A1c levels was strongly correlated with the amount of CPAP use (Babu et al., 2005). One observational cohort study examined the effect of CPAP on incident diabetes and found that among patients with moderate to severe OSA, the regular use of CPAP (determined by physician follow-up) was associated with a significant reduction of incident diabetes, even after adjusting for subsequent weight loss during an average of 2.7-year follow-up period (Botros et al., 2009).

In non-diabetic patients with OSA, numerous uncontrolled studies have examined the effects of CPAP treatment on prediabetic states such as glucose tolerance and insulin sensitivity. While some studies have reported positive findings (Harsch et al., 2004; Lindberg et al., 2006; Barcelo et al., 2008; Dorkova et al., 2008; Schahin et al., 2008; Cuhadaroglu et al., 2009; Henley et al., 2009; Oktay et al., 2009; Steiropoulos et al., 2009b; Mota et al., 2011; Tasali et al., 2011; Shpirer et al., 2012), others were negative (Saini et al., 1993; Cooper et al., 1995; Stoohs et al., 1996; Saarelainen et al., 1997; Ip et al., 2000; Smurra et al., 2001; Czupryniak et al., 2005; Trenell et al., 2007; Vgontzas et al., 2008; Carneiro et al., 2009; Comondore et al., 2009; Murri et al., 2009; Garcia et al., 2011). The majority of studies used the HOMA index (relying on fasting insulin and glucose levels) as a measure of insulin sensitivity (Czupryniak et al., 2005; Lindberg et al., 2006; Trenell et al., 2007; Barcelo et al., 2008; Dorkova et al., 2008; Carneiro et al., 2009; Comondore et al., 2009; Cuhadaroglu et al., 2009; Henley et al., 2009; Murri et al., 2009; Steiropoulos et al., 2009b; Tasali et al., 2011), while only a few studies used the gold standard hyperinsulinemic euglycemic clamp method (Saarelainen et al., 1997; Smurra et al., 2001; Harsch et al., 2004; Schahin et al., 2008) and one study used the ivGTT (Tasali et al., 2011). Three studies have used the standard OGTT method to assess post-challenge glucose tolerance (Smurra et al., 2001; Henley et al., 2009; Shpirer et al., 2012). The average CPAP treatment period ranged between one night and 6 months, with one study reporting findings after almost 3 years of follow-up (Schahin et al., 2008). Notably, only about half of the studies reported objective data on CPAP use and the overall reported average CPAP adherence was about 4–5 h/night. Most studies were conducted in obese men. In one study, CPAP improved insulin sensitivity (assessed by hyperinsulinemic euglycemic clamp) in 40 men even after 2 days of therapy (Harsch et al., 2004) and the improvement persisted at 3-months, particularly in those with a BMI less than 30 kg/m^2^. In contrast, an earlier study (Smurra et al., 2001) using the hyperinsulinemic euglycemic clamp and OGTT in 16 obese men found no change in insulin sensitivity or glucose tolerance after 2 months of CPAP. A few studies reported a positive effect of CPAP on insulin sensitivity in subgroups of patients, specifically those who used CPAP for more than 4 h/night (Dorkova et al., 2008; Steiropoulos et al., 2009a) or those with excessive daytime sleepiness (Barcelo et al., 2008). In one study, 8 weeks of CPAP treatment (average CPAP use ∼6.6 h/night) modestly improved insulin sensitivity (as assessed by ivGTT) in young women with polycystic ovarian syndrome despite morbid obesity (Tasali et al., 2011). Importantly, the change in insulin sensitivity correlated positively with CPAP use and negatively with BMI. In addition, both daytime and nighttime norepinephrine levels were decreased after CPAP and the reductions were greater with increased CPAP use, suggesting that the metabolic effects of CPAP therapy may be modulated by the hours of CPAP use and the degree of obesity (Tasali et al., 2011).

Overall, the uncontrolled studies that examined the effect of CPAP therapy on glucose metabolism have yielded mixed results. This could be partly explained by differences in patient population and techniques used to assess glucose metabolism as well as small sample size, poor adherence to CPAP, and potential changes in body composition during the studies.

### Evidence from randomized-controlled trials

To date, a total of nine studies using randomized-controlled designs have investigated the effects of CPAP therapy on measures of glucose metabolism (Table 4). All studies included sham-CPAP as the control group and most studies were conducted in men. The duration of intervention period varied between 1 week and 3 months. Some studies used a parallel group design (West et al., 2007; Lam et al., 2010; Nguyen et al., 2010; Kohler et al., 2011; Hoyos et al., 2012) in which patients were randomized to either CPAP or sham-CPAP, while others used a cross-over design (Coughlin et al., 2007; Sharma et al., 2011; Sivam et al., 2012; Weinstock et al., 2012) where patients were first randomized to CPAP or sham-CPAP, followed by the alternate therapy after a washout period. In six of the nine studies (Coughlin et al., 2007; West et al., 2007; Lam et al., 2010; Sharma et al., 2011; Hoyos et al., 2012; Weinstock et al., 2012), measures of glucose metabolism were used as primary outcome, whereas in the remaining three studies, fasting glucose (Nguyen et al., 2010; Sivam et al., 2012) and HOMA index (Kohler et al., 2011) were included as secondary outcomes. Overall, only three of the nine studies (Lam et al., 2010; Sharma et al., 2011; Weinstock et al., 2012) reported positive findings during the randomized treatment period. One study (Hoyos et al., 2012) had positive findings only in the open, non-randomized extended portion of CPAP therapy. Nightly treatment use (CPAP or sham-CPAP) was 5 h or less in most studies (Coughlin et al., 2007; West et al., 2007; Nguyen et al., 2010; Sharma et al., 2011; Hoyos et al., 2012; Sivam et al., 2012; Weinstock et al., 2012) except about 6 h in one study (Kohler et al., 2011), which *a priori* included patients who had been treated with CPAP for more than 12 months with an average compliance of 4 h/night. In another study with only 1 week of intervention, CPAP use, but not sham-CPAP, averaged about 6 h (Lam et al., 2010). Sample characteristics, OSA definition, measures of glucose metabolism, randomized treatment duration, nightly treatment hours, and main findings on glucose metabolism and other key outcomes from the nine randomized-controlled trials are summarized in Table 4.

**Table 4:** **Randomized-controlled studies examining the effects of continuous positive airway pressure (CPAP) treatment of OSA on glucose metabolism**.

Author/year	Sample	OSA definition	Measures ofglucose metabolism	Randomized treatment duration	Nightly treatment (h)	Main findings	
						Glucose metabolism	Other
Coughlin et al. (2007)	17 (CPAP/Sham) 17 (Sham/CPAP)	AHI >15	SI (HOMA), fasting glucose, and insulin	6 weeks	CPAP ∼3.9 h Sham ∼2.6 h	No difference in SI or fasting glucose and insulin	Improvement in waking blood pressure and baroreceptor sensitivity
West et al. (2007)	Type 2 diabetics 20 (CPAP) 22 (Sham)	ODI 4% ≥10	HbA1c, SI (euglycemic clamp, HOMA)	3 months	CPAP ∼3.3 h Sham ∼3.5 h	No difference in SI or HbA1c	Improvement in daytime sleepiness
Lam et al. (2010)	31 (CPAP) 30 (Sham)	AHI ≥15	SI (Short Insulin Tolerance Test)	1 week	CPAP ∼6.2 h Sham ∼4.5 h	Improvement in SI at 1 week Improvement in SI persisted in obese patients only at 12 weeks (non-randomized)	Improvement in blood pressure, lipids, and urinary catecholamine at 12 weeks (non-randomized)
Nguyen et al. (2010)	10 (CPAP) 10 (Sham-CPAP)	RDI ≥15	Fasting glucose	3 months	CPAP ∼5.1 h Sham ∼4.9 h	No difference in fasting glucose	Improvement in myocardial perfusion reserve and endothelial-dependent vasodilation
Sharma et al. (2011)	Metabolic syndrome 43 (CPAP/Sham) 43 (Sham/CPAP)	AHI ≥5	SI (HOMA), HbA1c, fasting glucose, and insulin	3 months	CPAP∼ 5.1 h Sham ∼4.8 h	Improvement in HbA1c No difference in SI or fasting glucose and insulin	Reversal of metabolic syndrome (13%) Improvement in blood pressure, lipids Reduced BMI, visceral, and subcutaneous fat
Kohler et al. (2011)	20 (CPAP) 20 (CPAP withdrawal)	ODI 4% ≥10	SI (HOMA)	2 weeks	CPAP ∼6.2 h Sham ∼6.0 h	No difference in SI	Increased sleepiness, blood pressure, and urinary catecholamines after CPAP withdrawal Impaired endothelial function after CPAP withdrawal No difference in lipids or psychomotor performance
Hoyos et al. (2012)	34 (CPAP) 31 (Sham)	AHI ≥20/h ODI 3% ≥15	SI (OGTT), Disposition index	12 weeks	CPAP ∼3.6 h Sham ∼2.8 h	No difference in SI at 12 weeks Improvement in SI at 24 weeks (non-randomized)	No difference in visceral fat or liver fat at 12 and 24 weeks
Sivam et al. (2012)	27 (CPAP/Sham or Sham/CPAP)	AHI ≥25/h ODI 3% ≥20	Fasting glucose	8 weeks	CPAP∼ 4.6 h Sham ∼3.4 h	No difference in fasting glucose	No difference in subcutaneous or visceral fat or liver fat
Weinstock et al. (2012)	Prediabetics (IGT) 25 (CPAP/Sham) 25 (Sham/CPAP)	AHI >15	SI (OGTT, Gutt index, HOMA) and presence of IGT	8 weeks	CPAP∼ 4.8 h Sham ∼3.4 h	No reversal of IGT No difference in HOMA or 2 h glucose Improvement in SI (Gutt index) and 2 h insulin only in severe OSA	N/A

*HOMA, homeostatic model assessment; HbA1c, hemoglobin A1c; OGTT, oral glucose tolerance test; IGT, impaired glucose tolerance; SI, insulin sensitivity; RDI, respiratory disturbance index*.

West et al. (2007) studied 42 obese patients with type 2 diabetes in a double-blind parallel-design and found no effect of CPAP on A1c levels or insulin sensitivity (estimated by the euglycemic clamp method), but reported significant improvements in daytime sleepiness (assessed by the Epworth Sleepiness Scale and the Maintenance Wakefulness Test) after 3 months of CPAP. Body composition including BMI, percent body fat (by bioimpedance technique), waist-hip ratio, and neck size did not change with treatment. In this study, the average nightly CPAP use was only about 3.3 h and no correlation was reported between CPAP compliance and measures of insulin resistance and A1c (West et al., 2007).

Coughlin et al. (2007) investigated the effect of 6 weeks of CPAP versus sham-CPAP in a cross-over design on cardiovascular and metabolic outcomes in 34 obese (mean BMI = 36.1 kg/m^2^) and non-diabetic men with symptomatic OSA. The authors found no significant difference in insulin sensitivity (as assessed by the HOMA index) or the presence of metabolic syndrome after CPAP use. Similar to the study by West et al. (2007), the average CPAP use was low, averaging about 3.9 h/night. Despite the relatively poor CPAP compliance, there was a significant improvement in blood pressure (Coughlin et al., 2007), suggesting that the amount and the duration needed to reverse cardiovascular and metabolic abnormalities may differ.

In a parallel-design study by Lam et al. (2010), 61 non-diabetic Chinese men with moderate to severe OSA and lesser degree of obesity (mean BMI = 27.5 kg/m^2^) were randomized to either CPAP or sham-CPAP for only 1 week. Those who were in the CPAP group were also re-assessed at 3 months. Insulin sensitivity was estimated by the short insulin tolerance test and the HOMA index. After 1 week of treatment, the CPAP group (about 6.2 h of average CPAP use) showed reduced insulin resistance and this improvement in insulin sensitivity was maintained at 3 months in those who were overweight (BMI of 25 or greater). After 3 months of CPAP use, there were improvements in blood pressure, plasma lipids, and urinary catecholamines, but no change was observed in the amount of visceral fat as measured by MRI (Lam et al., 2010).

In a small study with a parallel design, Nguyen et al. (2010) randomized 20 newly diagnosed, moderate to severe OSA patients to either CPAP or sham-CPAP for 3 months to investigate the impact of CPAP therapy on myocardial and endothelial function as main outcomes. While parameters of cardiovascular function improved after CPAP, there was no change in fasting glucose levels, which was included in the study as a secondary outcome.

In the largest trial, Sharma et al. (2011) studied 86 patients with severe OSA, of which the majority (87%) had metabolic syndrome and about 50% were diabetics. The patients were randomly assigned to 3 months of CPAP followed by 3 months of sham-CPAP, or vice versa, with a washout period of 1 month in between. The frequency of the metabolic syndrome was reduced after CPAP therapy with reversal found in 11 of 86 patients (13%) undergoing CPAP therapy versus 1 of 86 (1%) undergoing sham-CPAP. A1c was slightly but significantly improved after CPAP compared to sham-CPAP, but no significant differences were observed in fasting glucose and insulin levels or insulin resistance as assessed by HOMA index. The authors also observed small, but significant reductions in blood pressure, lipids, and BMI (−0.3 unit difference between CPAP versus sham-CPAP groups) as well as reductions in visceral and subcutaneous fat, quantified by computerized tomography.

Weinstock et al. (2012) randomized 50 patients (mean BMI = 39 kg/m^2^) with prediabetes and moderate to severe OSA to either 2 months of CPAP or sham-CPAP in a cross-over design with a 1-month washout period. Prior to randomization, each patient was enrolled in a 2-week run-in period, and only those who met minimal adherence criteria during the run-in period were then randomized. The primary outcome was normalization of IGT status as assessed by 2 h glucose levels during an OGTT. Although the study did not show that IGT normalizes after CPAP (average use was 4.8 h in the CPAP group), there was improvement in insulin sensitivity (assessed by the Gutt index) and 2 h insulin levels in a subgroup of patients with severe OSA who had an AHI of 30 or greater. Notably, for each hour of CPAP use, the mean change in insulin sensitivity from baseline increased by 4.2%. Interestingly, sleepy patients with an Epworth Sleepiness score of 16 or greater had larger improvements in insulin sensitivity, fasting insulin, and 2-h insulin as compared to non-sleepy subjects. This study also controlled for individual differences in sleep duration and visceral fat (as assessed by computerized tomography) in the analyses.

In the study by Kohler et al. (2011) 41 patients with OSA who were treated with CPAP were randomized to either CPAP withdrawal (sub-therapeutic CPAP), or continued CPAP, for 2 weeks. CPAP withdrawal resulted in rapid recurrence of OSA, increased subjective sleepiness, impaired endothelial function, increased urinary catecholamines, and elevated blood pressure and heart rate. However, there was no change in lipids or insulin sensitivity as estimated by the HOMA index.

Sivam et al. (2012) studied 27 patients who received both therapeutic and sham-CPAP in random order for 2 months with an intervening 1 month washout period. No difference was found in subcutaneous, visceral, and liver fat (primary outcomes) or fasting glucose levels (secondary outcome) between treatment arms.

The study by Hoyos et al. (2012) included 65 non-diabetic, obese men (mean BMI = 31.3 kg/m^2^) with moderate to severe OSA who were randomized to therapeutic or sham-CPAP for 3 months in a parallel group design. At the end of 3 months, all patients received therapeutic CPAP for an additional 3 months. The primary outcomes (from baseline to 12 weeks) were the change in visceral fat by computerized tomography and the change in insulin sensitivity based on OGTT. Although there were no differences in insulin sensitivity between the two groups after 12 weeks, the entire group using CPAP showed improved insulin sensitivity as compared to baseline at the end of 24 weeks during the non-randomized portion of the study (Hoyos et al., 2012). Of note, the therapeutic CPAP pressure was determined by an at-home titration, which may not be optimal, and the average CPAP use was only 3.6 h. Even though subgroup analyses were performed in “more compliant” patients (average use >4 h/night), there were only 5 out of 65 (8%) patients who used CPAP for more than 6 h.

In conclusion, there is still controversy as to whether CPAP treatment of OSA improves glucose metabolism. There is some evidence to support the hypothesis that the degree of obesity and the amount of CPAP use may be important factors in the metabolic response to CPAP. The current data also suggest that the effects of CPAP may vary according to the measured outcome. Large-scale randomized-controlled trials with robust assessments of insulin sensitivity and glucose tolerance will be required to fully determine the metabolic effects of CPAP. Moreover, future studies are also needed to investigate the optimal duration and the amount of CPAP use that is required to improve metabolic outcomes. Such interventional studies would be essential to address whether there is a causal link between OSA and alterations in glucose metabolism. Future studies should aim to determine subgroups of OSA patients who possess certain characteristics that make them more likely to get metabolic benefit from CPAP therapy. Identification of these metabolic phenotypes of OSA who are responsive to CPAP therapy could be a key factor in the clinical management and care of patients with prediabetes and type 2 diabetes.

## What are the Potential Mechanisms Underlying the Link between OSA and Insulin Resistance and Glucose Intolerance?

The two major characteristics of OSA, namely intermittent hypoxia and sleep fragmentation can lead to derangements in glucose metabolism by several mechanisms including activations of the sympathetic nervous system and hypothalamic-pituitary axis, and changes in the inflammatory pathways (Jun and Polotsky, 2009). Other putative mechanisms include hypoxic injury to pancreas and potential alterations in central (e.g., hypothalamic) pathways for glucose control. Animal models mimicking OSA have been used to elucidate the potential mechanisms that may be responsible for metabolic dysregulation in OSA. Intermittent hypoxia stimuli of varying frequency and severity were used in rodents when they are most likely to be asleep. In one study, electroencephalogram tracings were recorded and showed that intermittent hypoxia stimuli were accompanied by arousals, suggesting that sleep fragmentation is also an intrinsic feature of these hypoxia models (Polotsky et al., 2006). In these models, intermittent hypoxia led to sympathetic activation and hypertension (Fletcher et al., 1992; Fletcher, 2003), insulin resistance in both lean (Iiyori et al., 2007) and obese (Polotsky et al., 2003) rodents, impaired glucose homeostasis (Yokoe et al., 2008), and pancreatic beta-cell proliferation (Yokoe et al., 2008; Xu et al., 2009) as well as apoptosis of beta-cells (Yokoe et al., 2008; Xu et al., 2009). In a study by Gharib et al. (2012) mice that were exposed to 6 h of sleep fragmentation by the movement of an automated bar showed insulin resistance and glucose intolerance as well as altered transcriptional profile of visceral adipocytes. In healthy humans, one study that used intermittent hypoxia selectively during sleep found that 2–4 weeks of exposure to intermittent hypoxia resulted in increased morning blood pressure (Tamisier et al., 2009), but glucose metabolism was not assessed. Another study exposed healthy subjects to 5 h of intermittent hypoxia or normoxia during wakefulness and reported a decreased insulin sensitivity estimated by ivGTT (Louis and Punjabi, 2009). Two studies exposed healthy subjects to sleep fragmentation using acoustic stimuli and examined the effects on glucose metabolism by ivGTT. In the first study by Tasali et al. (2008a), all night selective suppression of slow wave sleep for three consecutive nights resulted in an approximate 25% decrease in insulin sensitivity without adequate compensatory rise in insulin secretion, leading to reduced glucose tolerance and increased diabetes risk (i.e., decreased disposition index). In the second study, non-selective sleep fragmentation for two nights was associated with a decrease in insulin sensitivity and non-insulin dependent glucose disposal (Stamatakis and Punjabi, 2009). In contrast to the study by Tasali et al. the second study noted an increase in the acute insulin response to glucose, suggesting that the beta-cell function was preserved. Notably, the second study was also associated with marked reductions in slow wave sleep, whereas other sleep stages were only minimally affected. Both of these sleep fragmentation studies were associated with elevated cardiac sympathetic output (Tasali et al., 2008a; Stamatakis and Punjabi, 2009). Overall, data from animal and human models that mimic OSA support an adverse effect of OSA on insulin resistance, glucose intolerance, and increased diabetes risk. The relative contributions of intermittent hypoxia versus sleep fragmentation on cardiometabolic outcomes remain to be determined.

In summary, current evidence strongly supports an independent association between OSA and insulin resistance and glucose intolerance, but a causal link remains to be determined. A substantial proportion of patients with type 2 diabetes suffer from unrecognized OSA. Conversely, type 2 diabetes is more prevalent among patients with OSA compared to those without OSA. Thus, the role of OSA in the management of type 2 diabetes is in urgent need of further rigorous assessment. The question of whether OSA represents an independent risk for the development of prediabetes and type 2 diabetes over time remains to be investigated by large prospective studies. Notably, weight loss, if successfully achieved can improve both OSA and glucose control, and thus should be recommended systematically to all patients with OSA. There is still debate whether CPAP treatment of OSA improves glucose metabolism. Also, the sufficient amount and duration of CPAP that is required to improve metabolic outcomes remains to be determined. Large-scale randomized-controlled trials of CPAP treatment of OSA with well-validated assessments of insulin sensitivity and glucose tolerance are needed. Identification of phenotypes of OSA patients who show better metabolic response to CPAP therapy would be important.

## Conflict of Interest Statement

The authors declare that the research was conducted in the absence of any commercial or financial relationships that could be construed as a potential conflict of interest.
